# Supplementary material for: Stochastic variation in the initial phase of bacterial infection predicts the probability of survival in D. melanogaster
Source: eLife. 2017 Oct 12;6:e28298. doi: 10.7554/eLife.28298 (PMC5703640; doi:10.7554/eLife.28298)
Supplement: Supplementary file 1. [file elife-28298-supp1.docx]

**Supporting Information**

**Supplementary Material and Methods**

**Supplementary file 1:** Primer sequences used in the qPCR analysis.

| Target gene | Direction | Primer sequence |
| --- | --- | --- |
| Diptericin | forward | 5’ GCTGCGCAATCGCTTCTACT 3’ |
|  | reverse | 5’ TGGTGGAGTGGGCTTCATG 3’ |
| RpL32 | Forward | 5’ GACGCTTCAAGGGACAGTATCTG 3’ |
|  | reverse | 5’ AAACGCGGTTCTGCATGAG 3’ |
